# RNA sequencing and lipidomics uncovers novel pathomechanisms in recessive X-linked ichthyosis

**DOI:** 10.3389/fmolb.2023.1176802

**Published:** 2023-06-07

**Authors:** Farrell McGeoghan, Emanuela Camera, Miriam Maiellaro, Manasi Menon, Mei Huang, Priya Dewan, Stela Ziaj, Matthew P. Caley, Michael Donaldson, Anton J. Enright, Edel A. O’Toole

**Affiliations:** ^1^ Centre for Cell Biology and Cutaneous Research, Blizard Institute, Faculty of Medicine and Dentistry, Queen Mary University of London, London, United Kingdom; ^2^ Laboratory of Cutaneous Physiopathology, San Gallicano Dermatological Institute-IRCCS, Rome, Italy; ^3^ Department of Dermatology, Royal London Hospital, Barts Health NHS Trust, London, United Kingdom; ^4^ Senzo Health Limited, London, United Kingdom; ^5^ Department of Pathology, University of Cambridge, Cambridge, United Kingdom

**Keywords:** ichthyosis, skin barrier, steroid sulfatase, ceramides, lipidomics

## Abstract

Recessive X-linked ichthyosis (RXLI), a genetic disorder caused by deletion or point mutations of the steroid sulfatase (*STS*) gene, is the second most common form of ichthyosis. It is a disorder of keratinocyte cholesterol sulfate retention and the mechanism of extracutaneous phenotypes such as corneal opacities and attention deficit hyperactivity disorder are poorly understood. To understand the pathomechanisms of RXLI, the transcriptome of differentiated primary keratinocytes with STS knockdown was sequenced. The results were validated in a stable knockdown model of STS, to confirm STS specificity, and in RXLI skin. The results show that there was significantly reduced expression of genes related to epidermal differentiation and lipid metabolism, including ceramide and sphingolipid synthesis. In addition, there was significant downregulation of aldehyde dehydrogenase family members and the oxytocin receptor which have been linked to corneal transparency and behavioural disorders respectively, both of which are extracutaneous phenotypes of RXLI. These data provide a greater understanding of the causative mechanisms of RXLI’s cutaneous phenotype, and show that the keratinocyte transcriptome and lipidomics can give novel insights into the phenotype of patients with RXLI.

## Introduction

Recessive X-linked ichthyosis (RXLI) is a genetic disorder occurring in approximately 1:1500 males (Craig et al., 2010). The disease presents in the first year of life with scaling and erythema developing with time into brown polygonal scales most marked on the shins, neck and abdomen but generally sparing the palmoplantar skin and flexures. There are often extracutaneous features, which include asymptomatic corneal opacities, neurological features (autism-related traits and attention deficit hyperactivity disorder (ADHD)) and hypogonadism (Crane and Paller, 2022). In an Italian study, ADHD was reported in 9 out of 33 RXLI patients (Diociaiuti et al., 2019). A study of female carriers showed increased rates of autism-related traits and post-partum depression compared to controls (Cavenagh, Chatterjee, and Davies, 2019).

A recent study using UK Biobank identified an association with atrial arrhythmias, haemorrhage after surgery and palmar fascial fibromatosis (Brcic et al., 2020), (Brcic et al., 2022). An online survey showed self-reported abnormal heart rhythms in 28%–35% of male and female carriers of Xp22.31 deletions and suggested that cardiac screening might be important (Wren et al., 2022).

The causative gene steroid sulfatase (STS) encodes a protein which hydrolyses cholesterol sulfate (CSO_4_) to cholesterol (Koppe et al., 1978) (Tiepolo et al., 1980; Yen et al., 1987). In the epidermis this occurs in the upper granular layer and stratum corneum, where STS activity increases up to 20-fold compared to the basal layer (Williams et al., 1983). In RXLI there is a total loss of STS activity in all layers of the epidermis, leading to adverse effects on the lipid content, delayed desquamation and acanthosis which is thought to result from CSO_4_ levels increasing up to 20-fold. In addition, mothers giving birth to babies with RXLI often experience prolonged labour (Paige et al., 1994).

Previous research *in vitro* has indicated CSO_4_ can affect the activity and expression of transglutaminase (TGM) 1, a cross-linking enzyme important for the formation of the cornified envelope (CE) and the cornified lipid envelope (CLE) (Nemes et al., 2000). Analysis of RNA sequencing of human keratinocyte HaCaT cells treated with cholesterol and cholesterol sulfate identified increased Yippee-like 3 (*YPEL3*), a gene expected to affect keratinocyte differentiation. Increased expression of YPEL3 in STS-deficient cell lines promoted cellular senescence and increased involucrin and loricrin (Baek et al., 2021). In addition, CSO_4_ has been shown *in vitro* to be a serine protease inhibitor, so it is theorised there is inhibition of kallikreins (KLK) 5 and 7 in RXLI, which would prevent desquamation (Sato et al., 1998). Thus far, these data have not been confirmed in RXLI samples and there is no data on the pathogenesis of extracutaneous signs, other than in those patients with contiguous deletions.

Here, we confirm reduced TGM1 expression and activity *in vivo* using RXLI skin samples and show upregulated expression of desmoglein-1 and reduced expression of KLK5. Furthermore, we report the results of transcriptome sequencing from primary keratinocytes with STS knockdown and validation in an STS knockdown model and RXLI skin. Immunostaining showed reduced epidermal expression of UDP-glucose ceramide glycosyltransferase (UGCG) and alkaline ceramidase 1 (ACER1), two important components of the ceramide synthesis pathway. Corneal opacities were linked to aldehyde dehydrogenase 1 family member A1 (ALDH1A1) and aldehyde dehydrogenase 3 family member A1 (ALDH3A1) downregulation, both of which are involved in corneal transparency (Lassen et al., 2007). A reduction was seen in oxytocin receptor (OXTR) expression which has been linked to behavioural disorders and prolonged labour (Fuchs et al., 1982) (Jacob et al., 2007). These data provide novel explanations for the development of extracutaneous features in RXLI, and further the understanding of how CSO_4_ and STS interact and regulate epidermal differentiation and lipid synthesis.

## Results

### RNA sequencing in primary keratinocytes with siRNA knockdown of STS

We analysed a data set of triple biological replicates from primary keratinocytes with siRNA knockdown of STS. The data discussed in this publication have been deposited in NCBI’s Gene Expression Omnibus and are accessible through GEO Series accession number GSE232622 (Barrett et al., 2013). After applying a cut-off point of *p* < 0.05 there were 3,454 upregulated and 3,608 downregulated genes in the transcriptome data set. Genes with log-fold change >2 were inputted into the Gene Ontology GO Enrichment Analysis using the PANTHER Classification System to identify enriched biological processes. Table 1 shows the top 20 downregulated biological processes. Of interest, these included enrichment of genes involved in cholesterol efflux (4.9-fold), astrocyte development (4.55-fold), keratinization (4.22-fold), keratinocyte differentiation (3.7-fold) and cardiac septum morphogenesis (3.19-fold). Table 2 shows the top 20 upregulated biological processes. This included enrichment of genes involved in negative regulation of epithelial cell differentiation (6.2-fold), cell substrate junction assembly and organisation (5.53 and 5.39-fold, respectively) and response to oestrogen (4.22-fold).

**TABLE 1:** Gene Ontology annotation of biological processes downregulated in keratinocytes with siRNA knockdown of STS. FDR = False discovery rate. Expected = expected number of genes downregulated. Fold-enrichment- Actual number of genes downregulated/expected.

GO biological processes	Reference list	Downregulated (1292)	Expected	Fold-enrichment	Raw *p*-value	FDR
antigen processing and presentation of endogenous peptide antigen (GO:0002483)	19	8	1.19	6.71	1.17E-04	1.06E-02
response to interferon-alpha (GO:0035455)	23	8	1.44	5.54	3.36E-04	2.39E-02
neural tube patterning (GO:0021532)	33	11	2.07	5.31	3.67E-05	4.39E-03
positive regulation of cholesterol efflux (GO:0010875)	26	8	1.63	4.9	6.61E-04	3.95E-02
antigen processing and presentation of endogenous antigen (GO:0019883)	26	8	1.63	4.9	6.61E-04	3.94E-02
astrocyte development (GO:0014002)	35	10	2.2	4.55	2.38E-04	1.90E-02
keratinization (GO:0031424)	83	22	5.21	4.22	1.68E-07	6.44E-05
ventricular septum morphogenesis (GO:0060412)	41	10	2.57	3.89	6.90E-04	4.05E-02
keratinocyte differentiation (GO:0030216)	138	32	8.66	3.7	4.75E-09	3.39E-06
intermediate filament organization (GO:0045109)	70	16	4.39	3.64	3.81E-05	4.49E-03
negative regulation of viral genome replication (GO:0045071)	57	13	3.58	3.63	2.03E-04	1.66E-02
SMAD protein signal transduction (GO:0060395)	63	14	3.95	3.54	1.48E-04	1.31E-02
embryonic digit morphogenesis (GO:0042733)	59	13	3.7	3.51	2.71E-04	2.03E-02
roof of mouth development (GO:0060021)	92	20	5.77	3.46	8.17E-06	1.41E-03
aorta development (GO:0035904)	56	12	3.51	3.41	5.73E-04	3.54E-02
epidermal cell differentiation (GO:0009913)	201	42	12.61	3.33	3.18E-10	2.94E-07
BMP signaling pathway (GO:0030509)	91	19	5.71	3.33	2.23E-05	3.09E-03
cardiac septum morphogenesis (GO:0060411)	70	14	4.39	3.19	3.80E-04	2.61E-02
intermediate filament cytoskeleton organization (GO:0045104)	92	18	5.77	3.12	7.58E-05	7.62E-03
intermediate filament-based process (GO:0045103)	93	18	5.84	3.08	8.56E-05	8.28E-03

**TABLE 2:** Gene Ontology annotation of biological processes upregulated in keratinocytes with siRNA knockdown of STS. FDR = False discovery rate.

GO biological processes	Reference list	Upregulated (745)	Expected	Fold-enrichment	Raw *p*-value	FDR
endoderm formation (GO:0001706)	54	13	1.95	6.65	4.61E-07	3.28E-04
endodermal cell differentiation (GO:0035987)	44	10	1.59	6.28	1.51E-05	3.70E-03
negative regulation of epithelial cell differentiation (GO:0030857)	49	11	1.77	6.2	6.29E-06	1.97E-03
regulation of cell migration involved in sprouting angiogenesis (GO:0090049)	38	8	1.38	5.82	1.71E-04	2.34E-02
regulation of transforming growth factor beta production (GO:0071634)	39	8	1.41	5.67	2.00E-04	2.64E-02
cell-substrate junction assembly (GO:0007044)	40	8	1.45	5.53	2.33E-04	2.97E-02
regulation of blood coagulation (GO:0030193)	70	14	2.53	5.53	1.17E-06	5.56E-04
negative regulation of blood coagulation (GO:0030195)	46	9	1.66	5.41	1.11E-04	1.70E-02
cell-substrate junction organization (GO:0150115)	41	8	1.48	5.39	2.70E-04	3.36E-02
regulation of hemostasis (GO:1900046)	72	14	2.61	5.37	1.57E-06	6.84E-04
negative regulation of hemostasis (GO:1900047)	47	9	1.7	5.29	1.28E-04	1.86E-02
regulation of coagulation (GO:0050818)	75	14	2.71	5.16	2.40E-06	9.40E-04
negative regulation of coagulation (GO:0050819)	50	9	1.81	4.97	1.93E-04	2.62E-02
positive regulation of blood vessel endothelial cell migration (GO:0043536)	54	9	1.95	4.61	3.22E-04	3.88E-02
regulation of wound healing (GO:0061041)	130	21	4.7	4.46	7.01E-08	8.45E-05
endoderm development (GO:0007492)	81	13	2.93	4.44	2.30E-05	5.08E-03
response to estrogen (GO:0043627)	72	11	2.61	4.22	1.43E-04	1.99E-02
negative regulation of wound healing (GO:0061045)	69	10	2.5	4.01	4.15E-04	4.72E-02
positive regulation of smooth muscle cell proliferation (GO:0048661)	85	12	3.08	3.9	1.42E-04	1.98E-02
regulation of ERBB signaling pathway (GO:1901184)	78	11	2.82	3.9	2.68E-04	3.36E-02

### Development of RXLI model and characterization of transglutaminase expression and desquamation

**FIGURE 1:**
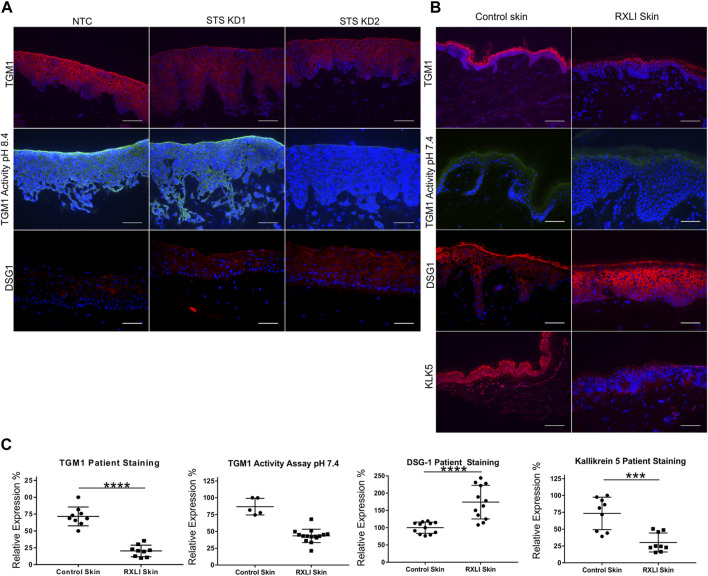
Loss of steroid sulfatase alters differentiation of the epidermis. **(A)** Immunohistochemistry of TGM1 expression in red (upper panel), TGM1 activity at pH 8.4 in green (middle panel) and DSG1 in red (lower panel) in 3D organotypics. **(B)** Immunohistochemistry of TGM1 expression (red) and activity at pH 7.4 (green), DSG1 (red) and KLK5 (green) expression in control skin and RXLI skin samples. DAPI (4′,6-diamidino-2-phenylindole) nuclear staining is blue. **(C)** Quantification of immunostaining shown in **(B)**. Three 40 X images were taken per sample (*n* = 3 for control and RXLI skin) for quantification of TGM1 and KLK5 expression. Five 40 X images were taken per sample (*n* = 1 for control skin and *n* = 3 for RXLI skin) for quantification of the TGM activity assay. Scale bar: 100 μm ^***^ *p* < 0.001 and ^****^ *p* < 0.0001 by unpaired 2-tailed *t*-test.

To validate genes of interest, skin biopsies were obtained from three RXLI patients. To confirm any changes observed were due to loss of STS, we also created using lentiviral particles STS shRNA knockdown (STS KD1 and STS KD2) and non-targeting control (NTC) cell lines in immortalised N/TERT keratinocytes. Knockdown was confirmed using qPCR showing greater KD in STS KD 2 (Supplementary Figure S1A). These cell lines were used to make a 3D model of RXLI (Supplementary Figure S1B). The 3D model of RXLI had decreased expression of TGM1 and decreased activity of TGM1 and TGM3 (pH 8.4) with a greater decrease in STS KD2 skin equivalents (Figure 1A). Decreased activity was also seen in the RXLI 3D model at pH 7.4 (Supplementary Figure S2), confirming that TGM1 activity alone was also reduced. In RXLI skin samples, there was a significant decrease of TGM1 expression and activity (Figures 1B, C). Desquamation requires a KLK cascade and CSO_4_ has been previously shown to inhibit KLK5 and KLK7 (Sato et al., 1998). We confirmed reduction of KLK5 in RXLI skin. KLK5 self-activates and then begins to activate other kallikreins, potentially making it the most important kallikrein for desquamation (Caubet et al., 2004). A reduction of KLK5 expression as observed here would likely affect the rest of the cascade, resulting in a reduction of several kallikreins required for desquamation resulting in the delay of desquamation seen in RXLI. Interestingly, loss of STS increased desmoglein 1 (*DSG1*) expression (Log Fold Change 1.049) in the RNA sequencing data set. Cell adhesion proteins are targets of KLKs including DSG1, desmocollin 1 (DSC1) and corneodesmosin (CDSN). The increase in DSG1 was confirmed by immunostaining in both the RXLI model (more obvious in STS KD2) in Figure 1A and in RXLI skin (Figures 1B, C), consistent with the reduced desquamation observed.

### Reduced expression of lipid metabolism enzymes in the RXLI model and skin

**FIGURE 2:**
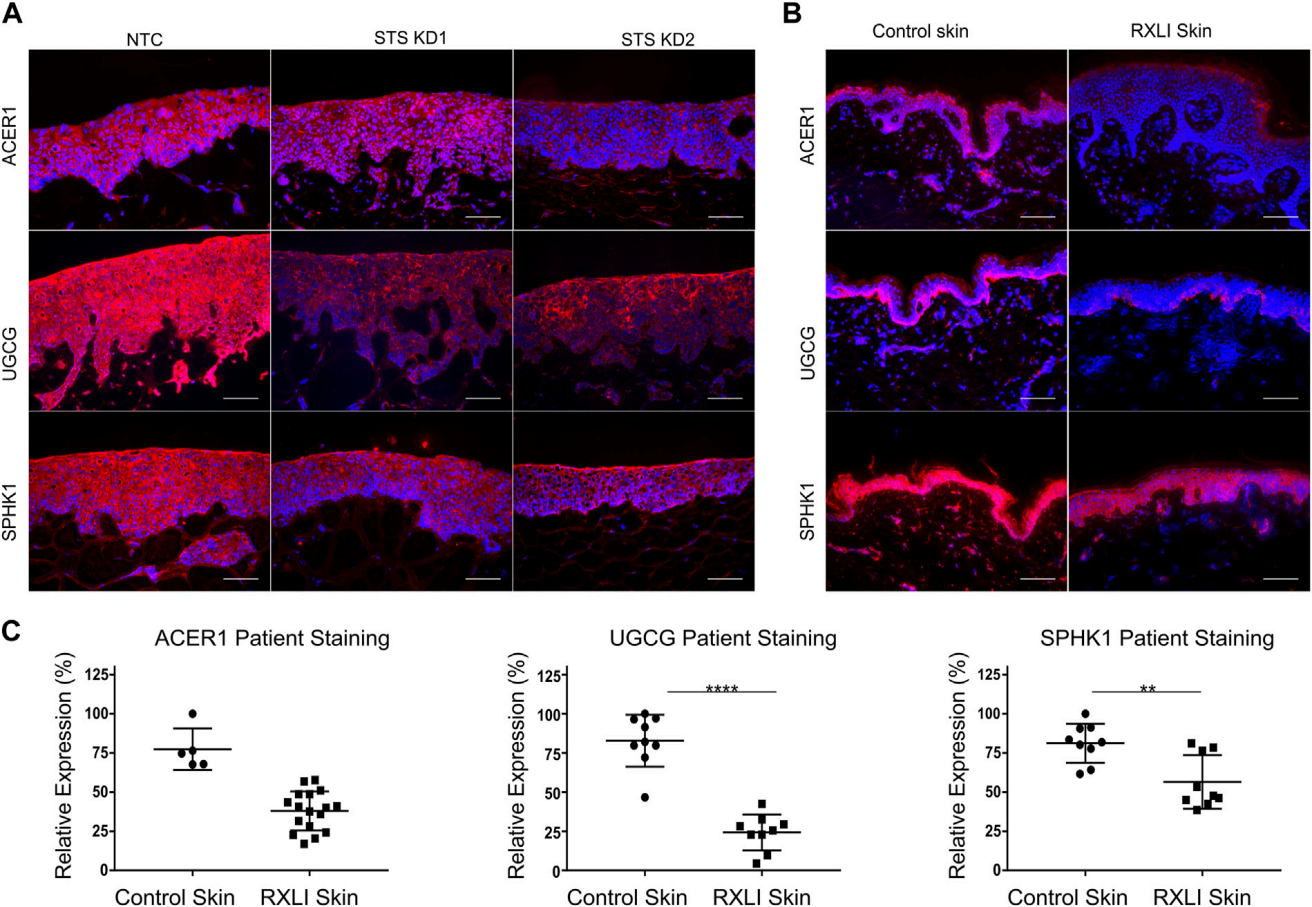
Loss of steroid sulfatase alters lipid metabolism in the epidermis. **(A)** Immunohistochemistry of ACER1, UGCG and SPHK1 expression in 3D organotypics. Scale bar: 100 µm **(B)** Immunohistochemistry of ACER1, UGCG and SPHK1 expression in control skin and RXLI skin samples. All antigens of interest are in red. DAPI nuclear staining is blue. **(C)** Quantification of immunostaining shown in **(B)**. For UGCG and SPHK1 quantification three 40 X images were taken per sample (*n* = 3 each for control and RXLI skin). For ACER1 five 40 X images were taken per sample (*n* = 1 for control skin and *n* = 3 for RXLI skin). Scale bar: 100 μm ^**^ *p* < 0.01 and ^****^ *p* < 0.0001 by unpaired 2-tailed *t*-test.

Differentially expressed genes in primary keratinocytes with STS knockdown included *ACER1* and *UGCG*, which encode enzymes important for lipid metabolism in the epidermis (Amen et al., 2013) (Sun et al., 2008). Alkaline ceramidase 1 hydrolyses long-chain ceramides to sphingosine, which is then phosphorylated to form sphingosine-1-phosphate, regulated by sphingosine 1 kinase (SPHK1). The UGCG enzyme catalyses the first step of glycosphingolipid production, synthesising glucosylceramides (GluCers) from ceramides and UDP-glucose. Loss of these enzymes could affect bioactive lipid content within the epidermis, so RXLI skin samples and the 3D model were immunostained for ACER1 and UGCG (Figures 2A, B). In the 3D model, expression of all 3 proteins was reduced in both STS KD models (Figure 2A). As previously shown, ACER1 was expressed in differentiated epidermis with a clear reduction in RXLI skin (see Figures 2B, C). The UGCG expression was strong in normal basal epidermis with weaker expression in the upper epidermis and was markedly decreased in RXLI skin (see Figures 2B, C). Expression of SPHK1 was seen throughout the epidermis in both the NTC 3D model and in normal control skin. A reduction was seen in both STS KD models and in RXLI epidermis (see Figures 2A–C).

### Reduced expression of aldehyde dehydrogenases and the oxytocin receptor in the RXLI model and skin

**FIGURE 3:**
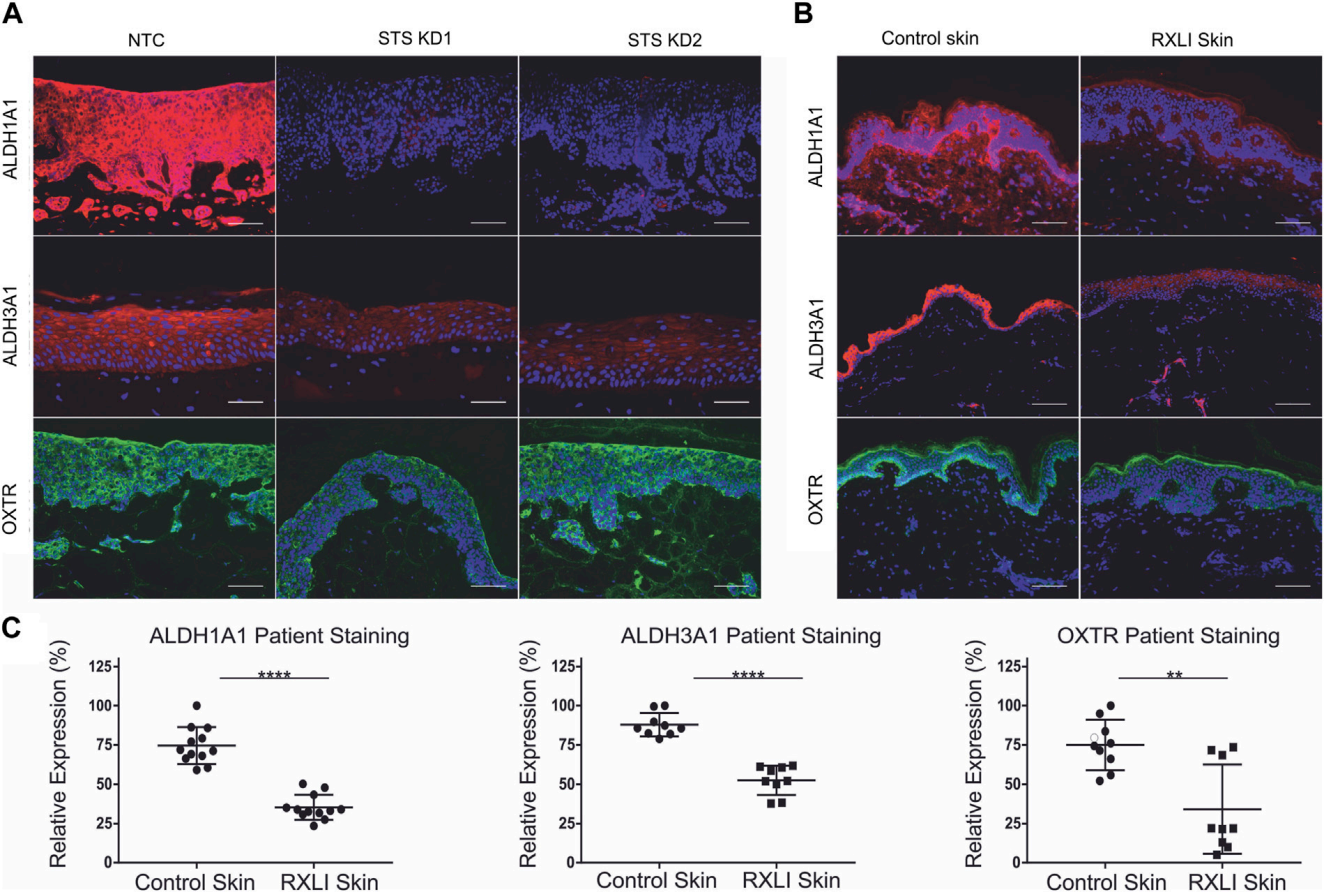
Loss of steroid sulfatase results in altered expression of genes linked to extracutaneous features of RXLI. **(A)** Immunohistochemistry of ALDH1A1 (red), ALDH3A1 (red) and OXTR (green) expression in 3D organotypics. Scale bar: 100 µm. **(B)** Immunohistochemistry of ALDH1A1 (red), ALDH3A1 (red) and OXTR (green) expression in control skin and RXLI skin samples. DAPI nuclear staining is blue. **(C)** Quantification of immunostaining shown in **(B)**. Three (ALDH3A1 and OXTR) or four (ALDH1A1) 40 X images were taken per sample (*n* = 3 each for control and RXLI skin) for quantification. Scale bar: 100 μm ^**^ *p* < 0.01 and ^****^ *p* < 0.0001 by unpaired 2-tailed *t*-test.

The transcriptome of primary keratinocytes with STS knockdown showed altered expression of *ALDH1A1*, *ALDH3A1* and *OXTR*. Investigation of the literature indicated they could be linked to extracutaneous features of RXLI. There is evidence that ALDH1A1 and ALDH3A1 are important for transparency of the cornea (Lassen et al., 2007; Arrowsmith and Wray, 2014), whereas OXTR has been linked to behavioural disorders and is known to regulate parturition (Rijlaarsdam et al., 2017) (Arrowsmith and Wray, 2014). Thus, immunostaining for ALDH1A1, ALDH3A1 and OXTR was performed in the RXLI model (Figure 3A), while the expression of ALDH1A1, ALDH3A1 and OXTR was also investigated in normal control skin and RXLI skin (Figure 3B). All three had decreased expression in the RXLI model compared to NTC (Figure 3A). ALDH1A1 was expressed in the stratum corneum and basal epidermis close to the basement membrane in control skin and markedly reduced expression was observed in RXLI skin (Figures 3B, C). ALDH3A1 was expressed in suprabasal epidermis in normal skin and was markedly reduced in RXLI skin. OXTR expression was present in the basal epidermis in normal skin and was markedly decreased in RXLI skin (Figure 3B).

### Lipidomic analysis of the RXLI model

**FIGURE 4:**
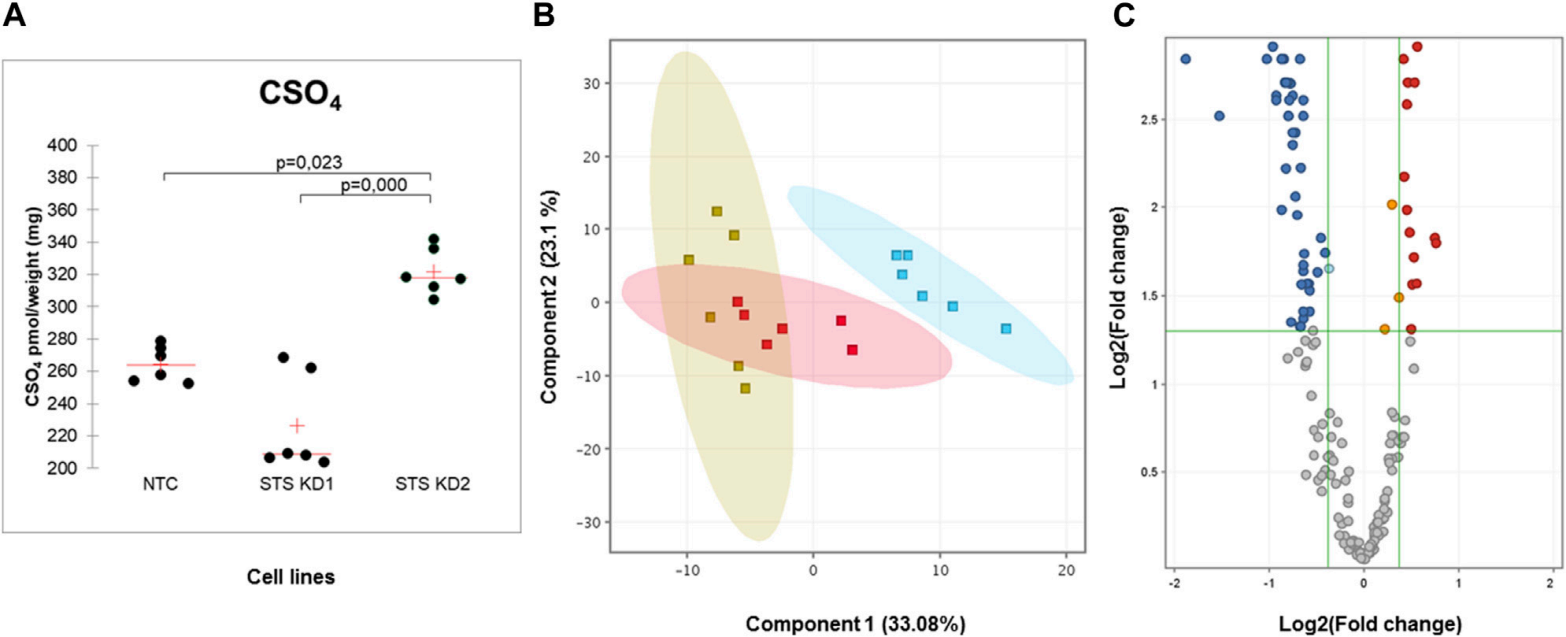
Retention of cholesterol sulfate and altered lipidome of RXLI model. **(A)** Quantitative assessment (LCMS) of cholesterol sulfate (CSO_4_) in NTC, STS KD1, and STS KD2. Quantitative amounts are reported as pmol/weight (mg) of the 3D organotypics. **(B)** Principal components analysis (PCA) of the lipids detected in NTC (red), STS KD1 (yellow) and STS KD2 (blue) organotypics. **(C)** Volcano plot indicating the changes in the lipid abundance in STS KD2 compared to control (NTC). Each dot represents one lipid species. Differential abundance is reported as log2 transformed fold change (FC) vs. NTC. Lipid species diminished or increased at a statistically different level were indicated with blue and red dots, respectively. Identities, molecular mass, and regulation of species that passed significance = 0.05 and FC = 1.3 cut-offs are reported in Table 3. Upregulated and downregulated species that did not pass the significance cut-off of 0.05 are displayed in orange and light blue, respectively.

In order to understand the effect of STS knockdown further, changes in lipid profiles induced in the RXLI model were analysed using lipidomics as described in the Methods. We initially applied an untargeted approach to the whole lipid extracts from 3D epidermis. Only entities whose abundance appeared to be affected by the STS knockdown at a statistically significant extent were further processed for their identification. As shown in Figure 4A, CSO_4_ expression was significantly increased in STS KD 2 epidermis, validating the model as CSO_4_ retention is seen in RXLI. Principal component analysis (see Figure 4B) showed that there was a high degree of overlap between NTC and STS KD1, while there was an appreciable difference between NTC and STS KD2. The volcano plot in Figure 4C represents the changes of lipid levels in STS KD2 compared to NTC. Table 3 shows detailed changes that satisfied the cut-offs of fold change >1.4 and significance ≤0.05 in regulation of lipid species in STS KD2 versus NTC, as depicted in the volcano plot in Figure 4C. Supplementary Figure S3 shows hierarchical clustering of lipids modulated in STS KD1 and KD2 compared to NTC again confirming major changes in STS KD2 (lipid species are detailed in Supplementary Table S4). Several ceramide species belonging to the non-hydroxy fatty acyl dihydrosphingosine (NDS) subclass class consisting of non-hydroxy fatty acids (N) and dihydrosphingosine base (DS), which is also known as sphinganine, were upregulated. Ceramide nomenclature is as described in these references (Masukawa et al., 2008; Liebisch et al., 2020). The increase of these ceramides may reflect a promoted *de novo* synthesis of ceramides or defective desaturation to the sphingosine base by sphingolipid desaturases, such as DEGS1. One member of the non-hydroxy fatty acyl sphingosine (NS) ceramides was upregulated. Ceramides containing deoxysphinganine as the sphingoid base were also upregulated. The increase of these anomalous ceramides may be due to condensation of alanine (instead of serine) with palmitoyl-CoA (Muthusamy et al., 2020). Several members of the tri hexosyl ceramides (Hex3Cer) were downregulated in KD2. Decreased levels of these sphingolipid metabolites in KD2 indicates deregulated sphingolipid synthesis/metabolism and trafficking (Iwabuchi et al., 2015). Moreover, the significantly decreased levels of N-acylphosphatidylethanolamine (NAPE) suggests deranged metabolism of endocannabinoids which may be related to the decrease of phosphatidylethanolamine (PEs) species (Coulon et al., 2012). The significance of a moderate, but statistically significant, decrease of etherPEs requires further investigation (Fontaine et al., 2020).

**TABLE 3:** Lipid species upregulated and downregulated in STS KD2 cells compared to control.

Compound	Mass	FC (STS KD2 Vs. NTC)	Log FC (STS KD2 Vs. NTC)	Regulation (STS KD2 Vs. NTC)
Cholesterol sulfate	466,3115	1,49	0,57	up
Cer(d18:0/22:0)	623,6191	1,45	0,53	up
Cer(d18:0/24:0)	651,6520	1,40	0,49	up
Cer(d18:0/26:0)	679,6835	1,38	0,46	up
Cer(d18:0/28:0)	707,7141	1,34	0,42	up
Cer(d18:1/22:0)	621,6017	1,42	0,51	up
Cer(m18:0/20:0)	579,5908	1,42	0,50	up
Cer(m18:0/22:0)	607,6240	1,48	0,56	up
Cer(m18:0/24:0)	635,6570	1,46	0,54	up
Cer(m18:0/24:1)	633,6417	1,37	0,46	up
Cer(m18:0/26:0)	663,6881	1,34	0,43	up
Cer(m18:0/26:1)	661,6730	1,37	0,46	up
EtherPC 40:2	827,6639	1,69	0,75	up
SM 40:2;O2	784,6430	1,70	0,76	up
NAPE(48:2)	925,7069	−1,56	−0,64	down
Hex3Cer(d18:0/14:1)	995,6363	−1,32	−0,41	down
Hex3Cer(d18:0/16:1)	1023,670	−1,71	−0,78	down
Hex3Cer(d18:0/18:1)	1051,699	−1,48	−0,57	down
Hex3Cer(d18:0/20:1)	1079,731	−1,48	−0,57	down
Hex3Cer(d18:0/24:1)	1135,794	−1,60	−0,68	down
Hex3Cer(d18:1/14:0)	995,6363	−1,36	−0,45	down
Hex3Cer(d18:1/16:0)	1023,670	−1,71	−0,77	down
Hex3Cer(d18:1/18:0)	1051,699	−1,56	−0,64	down
Hex3Cer(d18:1/18:1)	1023,670	−1,71	−0,78	down
Hex3Cer(d18:1/20:0)	1079,730	−1,50	−0,58	down
Hex3Cer(d18:1/20:1)	1051,699	−1,54	−0,63	down
Hex3Cer(d18:1/24:0)	1135,794	−1,59	−0,66	down
Hex3Cer(d18:1/24:1)	1133,778	−1,82	−0,87	down
Hex3Cer(d18:1/26:1)	1161,809	−1,70	−0,76	down
PE 31:2	673,4664	−1,58	−0,66	down
PE 32:1	689,5011	−1,60	−0,67	down
PE 32:0	691,5161	−1,55	−0,63	down
PE 34:4	711,4799	−1,56	−0,64	down
PE 34:3	713,5015	−1,62	−0,70	down
PE 34:2	715,5223	−1,89	−0,92	down
PE 34:1	717,5291	−1,94	−0,96	down
PE 35:5	727,5234	−1,51	−0,59	down
PE 36:4	739,5121	−1,73	−0,79	down
PE 36:3	741,5337	−1,68	−0,75	down
PE 36:2	743,5466	−2,03	−1,02	down
PE 36:1	745,5616	−1,89	−0,92	down
PE 38:4	767,5443	−1,71	−0,78	down
PE 38:3	769,5597	−1,77	−0,82	down
PE 38:2	771,5786	−1,78	−0,83	down
PE 40:8	787,5107	−1,55	−0,63	down
PE 40:7	789,5289	−1,68	−0,75	down
PE 40:6	791,5450	−1,79	−0,84	down
EtherPE 32:2	673,5048	−1,55	−0,63	down
EtherPE 34:3	699,5168	−1,65	−0,72	down
EtherPE 34:2	701,5359	−1,57	−0,65	down
EtherPE 36:6	721,5056	−1,82	−0,87	down
EtherPE 36:5	723,5194	−1,74	−0,80	down
EtherPE 36:4	725,5350	−1,64	−0,72	down
EtherPE 36:2	729,5682	−1,77	−0,82	down
EtherPE 38:6	749,5345	−1,77	−0,82	down
EtherPE 38:5	751,5484	−1,73	−0,79	down
EtherPE 40:7	775,5501	−1,68	−0,75	down
EtherPE 40:6	777,5673	−1,82	−0,86	down
SM 32:1;O2	674,5364	−1,40	−0,49	down

## Discussion

Apart from deletion of *STS* and retention of CSO_4_, little is known about the underlying pathomechanisms in RXLI, or how extracutaneous phenotypes develop. These data further the understanding of RXLI pathomechanisms in skin, by showing loss of STS results in decreased expression of TGM1, KLK5, ACER1, UGCG, SPHK1 and decreased TGM activity. Taken together, these data suggest STS is important for regulating epidermal homeostasis, lipid metabolism, proliferation, differentiation, and desquamation. The data presented also provide insights into pathomechanisms involved in corneal opacities, behavioural changes and prolonged labour in female carriers of RXLI. Although, we have not explored further, the presence of a gene cluster involved in cardiac septum morphogenesis may be relevant to the development of arrhythmias in some RXLI patients.

Mice with *Acer1* knockout have increased levels of ceramides and sphingomyelin, and decreased levels of sphingosine and sphingosine-1-phosphate (S1P). Furthermore, the mice have dry, hyperplastic skin and a thickened stratum corneum (Liakath-Ali et al., 2016). The phenotype of these mice is similar to that of RXLI, particularly the dry skin and thickened stratum corneum, which suggests loss of ACER1 contributes to RXLI skin pathology. The activity of SPHK1 is crucial for generating S1P, which has anti-proliferative effects on keratinocytes (Di Nardo et al., 2000). Less SPHK1 will generate less S1P, indicating the loss of SPHK1 observed will result in a proliferative phenotype and altered differentiation.

A knockout mouse model of *Ugcg* presented with dry skin, thickening of the epidermis and hyperkeratosis, all phenotypes of RXLI. The knockout mice had extended expression of K14 up through the suprabasal layers, and overexpression of K6, which would both indicate a proliferative phenotype (Amen et al., 2013) (Jennemann et al., 2007). Importantly, *Ugcg* knockout mice have reduced levels of free-extractable glucosylceramides and increased free-extractable ceramides. However, protein-bound ceramides are severely reduced, indicating UGCG is important for producing protein-bound ceramide precursors (protein-bound glucosylceramides). It is probable that the reduction of UGCG in RXLI would alter ceramide metabolism by lowering the glucosylceramide content of the epidermis, reflected by the decreased Hex3Cer shown in Table 3. As shown in mouse models, this would prevent the formation of protein bound ceramides, and impact on the cornified lipid envelope.

Both ALDH1A1 and ALDH3A1 are important in reducing UVR damage in the cornea and lens as their loss results in higher levels of ROS and thus damage to the eye. It is believed this is how the corneal opacities develop in *Aldh1a1/Aldh3a1* knockout mice (Lassen et al., 2007). It was also noted previously that knockdown of OXTR in keratinocytes and fibroblasts caused increased levels of ROS and decreased levels of glutathione, an important antioxidant (Deing et al., 2013). Therefore, loss of OXTR may also contribute to corneal opacities by further reducing the cornea’s protection against stressors such as ultraviolet radiation. With our confirmation of reduced ALDH1A1, ALDH3A1 and OXTR expression in RXLI, we propose this is a contributing factor to the corneal opacities. Interestingly, the majority of corneal opacities in RXLI patients do not develop until the second or third decade of life. Damage caused by UVR may accumulate over time and act in combination with loss of these proteins in the development of corneal opacities, explaining why these develop later in life. As only half of patients develop corneal opacities, there may be other genetic or environmental factors involved. The aldehyde dehydrogenase family members are important components of the retinoic acid signalling pathway which is important in regulating epithelial proliferation (Kumar et al., 2017). A reduction in ALDH1A1 and ALDH3A1 may also affect keratinocyte proliferation.

Behaviours on the autistic spectrum and ADHD are more common in RXLI patients (25% and 40%, respectively) than the general population (Kent et al., 2008; Deing et al., 2013). It has been shown that loss of STS due to point mutations alone can result in RXLI patients developing ADHD. Interestingly, dysregulation of ceramides, alkaline ceramidases and S1P have been implicated in neurodegenerative disorders, indicating the potentially altered sphingolipids in RXLI may contribute to the behavioural disorders associated with RXLI (Cutler et al., 2004; Ben-David and Futerman, 2010; Wang et al., 2015). There are now numerous studies which conclude that polymorphisms in the *OXTR* gene confer a risk of behavioural disorders (Jacob et al., 2007; Lerer et al., 2008; Liu et al., 2010). It is theorised this is a result of oxytocin being unable to properly elicit its neurological effects due to loss of its receptor.

In RXLI, reduced placental STS results in prolonged labour. Oxytocin is used to induce uterine contractility (Fuchs et al., 1982). A reduction of placental OXTR expression may contribute to prolonged labour. Transport of OXT has been shown to occur from mother to foetus and *vice versa*, which helps explain how loss of OXTR due to RXLI could potentially affect the mother and parturition regulation (Malek, Blann, and Mattison, 1996). This reduction of OXTR could be a result of decreased oestrogen, or a concurrent mechanism that also contributes to prolonged parturition.

In summary, in this paper, we show that loss of STS in human epidermis causes major changes to ceramide and sphingolipid synthesis. This expands our understanding of RXLI pathogenesis and has implications for the skin barrier. The changes in sphingolipids may also be associated with the neuropsychiatric symptoms experienced by these patients. The connection between ALDH1A1 and ALDH3A1 loss and corneal opacities, and OXTR loss and prolonged labour and behavioural disorders provides a new explanation for the extracutaneous features of RXLI. In addition, the RNA-Seq data showed altered RNA expression of several genes linked to behavioural disorders and brain and cardiac development which could be explored further. Further work could be performed in a mouse model or using patient-derived induced pluripotent stem cells which could be differentiated into cells of interest including cardiomyocytes or neurons. A better understanding of the biological mechanisms underlying RXLI may lead to new treatments in the future.

## Materials and methods

### Study approval

This study was conducted according to the Declaration of Helsinki Principles and was approved by the East London and City Health Authority Research Ethics Committee. Written informed consent was obtained from patients before samples were taken.

### Control skin samples and RXLI skin samples

Dermal fibroblasts were isolated from neonatal foreskins. Redundant normal skin was used as a control for RXLI skin samples. Samples of RXLI skin were obtained from 3 individuals diagnosed with RXLI, confirmed by either steroid sulfatase activity assays or genetic testing.

### Cell culture and Knockdown cell lines

The human keratinocyte telomerase reverse-transcriptase–immortalized (h/TERT-immortalized) N/TERT-1 cell line derived from clinically normal foreskin tissue and supplied by James Rheinwald (Department of Dermatology, Harvard University Medical School, Boston, Massachusetts, United States) (Dickson et al., 2000) was grown in RM + growth media (DMEM/F-12, 10% FBS, 1× penicillin streptomycin [P/S], 0.4 μg/mL hydrocortisone, 0.5 μg/mL insulin, 10 ng/mL epidermal growth factor, 0.1 nM cholera toxin, 5 μg/mL transferrin, 20 p.m. liothyronine) and incubated at 37°C, 5% CO2. Human primary fibroblasts isolated from fresh redundant skin were grown in fibroblast growth media (DMEM, 10% FBS, 1× P/S) and incubated at 37°C, 5% CO2. Primary keratinocytes were isolated from neonatal foreskin and grown in Epilife media. SMARTvector 2.0 lentiviral shRNA particles (GE Healthcare, UK) targeting exons five and ten were used to transduce N/TERT keratinocytes with an shRNA construct in order to generate a stable knockdown of STS (see Supplementary Table S1). A non-targeting shRNA construct was transduced into keratinocytes and used as a control. The construct included a GFP reporter to allow confirmation of successful transduction.

### 3D model of RXLI

Collagen and Matrigel gels were prepared by mixing 3.5 volumes of collagen I with 3.5 volumes of Matrigel, one volume of MEM, one volume of FBS and 1 volume of primary fibroblasts. 1 ml of gel mix was added per well required of a 24 well plate and incubated at 37°C for 1 h. Subsequently, 1 ml of media were added to the top of the gel and incubated overnight at 37°C. The media were aspirated from the gel, and N/TERT keratinocytes infected with shRNA lentiviral particles were seeded on top of the gel and incubated at 37°C overnight. The gels were then raised to the air/liquid interface on a steel grid placed in a 6 well plate. The gels were incubated for a further 14 days.

### Preparation of tissues

Tissues were fixed in 4% PFA and embedded in paraffin using an automated tissue processor. Sections (5 µm-thick) of 4% PFA-fixed and paraffin embedded samples were cut using a Reichert-Jung 2035 microtome and placed on SuperFrost Plus slides. Sections were then deparaffinized in xylene and then rehydrated through a graduated ethanol series and water before immunostaining. Cryosections (10 µm-thick) of frozen samples mounted in OCT (Tissue-Tek, NL) were cut in a cryostat, placed on SuperFrost Plus slides and stored at −80°C. When used, sections were left to air dry for 10 min then washed once in PBS before immunostaining.

### Immunostaining

Sections were washed in PBS for 5 min, then blocked by incubating in IFF (1% BSA w/v, 2% FBS v/v in PBS) containing 5%–10% goat serum for 1 hour. Sections were then probed with primary antibody diluted in PBS containing 1% BSA and incubated on the section at RT for 2 hours or overnight at 4°C. Sections were washed three times with PBS before addition of the secondary antibody, Alexa Fluor 568-red or 488-green, goat anti-rabbit or goat anti-mouse (Invitrogen, CA, United States) at a 1:500 dilution for 1 hour at room temperature. Sections were washed three times in PBS before DAPI at a 1:1000 dilution was used as a nuclear stain. Following a further three washes in PBS the sections were mounted using Immu-mount (Thermo Fisher Scientific MA, United States). Negative controls consisted of mouse or rabbit IgG diluted to the same concentration as the primary antibody. Images were obtained using a Leica MM epi-fluorescence microscope. Antibodies used are detailed in Supplementary Table S2.

### Transglutaminase assay

Cryosections were washed with PBS for 5 min. Four sections were used per sample. Sections were blocked by incubation in 0.1 M Tris with 3% BSA at either pH 7.4 or pH 8.4. Sections were then incubated with 0.1 M Tris with 3% BSA containing either 20 µl 10 mM biotinylated monodansylcadaverine +20 µl 0.5 M CaCl_2_ per ml (positive sample) or 20 µl 10 mM biotinylated monodansylcadaverine +20 µl 0.5 M EDTA per ml (negative sample) at either pH 7.4 or pH 8.4 for 1 hour. The reaction was stopped by incubation of sections for 10 min with PBS +20 µl 0.5 M EDTA per ml. The sections were then washed three times with PBS. Streptavidin-Alexa488 diluted 1:2000 and DAPI diluted 1:1000 in PBS was applied to the sections for 30 min. The sections were then washed a further three times and mounted using Immu-mount. Images were obtained using a Leica MM epi-fluorescence microscope.

### Quantification

The staining of RXLI patient and normal control skin was quantified using Cell Profiler (3.0.0) which measured the intensity of staining across each image taken. This allowed us to calculate an average intensity per cell for control skin samples and RXLI patient samples, which were then averaged across multiple images from the same section to produce an overall value.

### RNA work

Total RNA was extracted from cells in a six well plate and purified using the RNAeasy kit (Qiagen, NL) according to the manufacturer’s instructions. Concentration of RNA samples was determined by measuring the absorbance at 260 nm with a spectrophotometer after RNA extraction (Nanodrop, ND-1000 Spectrophotometer) and stored at −80°C. cDNA was synthesised from total RNA using SuperScript VILO cDNA synthesis kit (Invitrogen, CA, United States) according to the manufacturer’s instructions.

### qPCR

The qPCR was performed with 5 ng of cDNA using the KAPA SYBR FAST qPCR kit (Biosystems, ES). cDNA was mixed with 1x KAPA SYBR FAST qPCR Master Mix, forward primer (200 nM), reverse primer (200 nM), and carboxy-X-rhodamine (ROX) reference dye low. The mix was adjusted to a reaction volume of 10 μl with water. The qPCR reaction was carried out using the 7,500 Real Time PCR system (Life Technologies, CA, United States) with the following cycle sequence: initiation at 95°C for 5 min, then 40 cycles of melting at 95°C for 10 s, annealing at 60°C for 30 s, and extension at 72°C for 40 s. Each sample was analysed in triplicate and data analysis was performed using the 7,500 System Detection Software v1.4 (Life Technologies, CA, United States) and Microsoft Excel 2011. HPRT was used as an internal control. Primers used are detailed in Supplementary Table S3.

### RNA Sequencing

HISAT2 was used to align sequencing reads to a reference genome. This tool uses the Burrows-Wheeler transform and the FM index to align reads with Bowtie2 as the algorithmic core. Once the reads are aligned, HTSeq is used to produce counts for each gene of how many aligned reads overlap its exons. This preprocesses the RNA-Seq data for differential expression analysis. The counts are fed into DESeq2, a tool used for differential expression analysis based on a model using negative binomial distribution.

### Statistics

Statistical analysis was determined by a Student’s unpaired two-tailed *t*-test using GraphPad Prism 7.03 (GraphPad Software). A *p*-value of 0.05 or less was considered statistically significant.

### Sample processing for the analysis of lipids

Organotypics (NTC, n = 3, STS KD1 n = 3, and STS KD2 n = 3) were treated as a whole. Organotypics were weighed and lipids extracted with a chloroform/methanol mixture 2:1 after addition of the internal standard mixture containing SPLASH Lipidomix^®^, LM6002, and d31CerNS (Avanti Polar Lipids, United States), and in-house mixed deuterated standards supplied by C/D/N isotopes and Toronto Research Chemicals, both from Canada. Aliquots of dissolved lipid extracts were analysed in duplicate by GCMS for the quantification of cholesterol, desmosterol, and free fatty acids. The dissolved lipid extracts were further analysed in duplicate by untargeted LCMS in positive and negative ion mode. The results of the untargeted approach were normalised by the internal standard d31CerNS and the weight of each sample. Quantitative results from both GCMS and LCMS were normalised by the mg of tissue weight and reported as pmol/weight (mg of tissue).

### Gas chromatography-mass spectrometry

Gas chromatography coupled to electron ionisation mass spectrometry (GCMS) dual scan-selected ion monitoring was employed to determine target compounds in the lipid extracts. Samples were analysed with a GC 7890 A coupled to the MS 5975 VL analyzer (Agilent Technologies, CA, United States). Analysis of free cholesterol was performed simultaneously to free fatty acids (FFAs) analysis by a GCMS method as previously reported (Singh et al., 2018). 20 µL of the lipid extracts dissolved in 200 µL of CHCl_3_/MeOH 2:1 mixture were dried under nitrogen and derivatized with 50 µL BSTFA added with 1% trimethylchlorosilane (TCMS) in pyridine to generate the trimethylsilyl (TMS) derivatives. The reaction was carried out at 60°C for 60 min. GC separation was performed with the 30 m–0.250 (i.d.) GC DB-5MS UI fused silica column (Agilent Technologies, CA, United States), chemically bonded with a 5% diphenyl 95% dimethylpolysiloxane cross-linked stationary phase (0.25 mm film thickness). Helium was used as the carrier gas. Samples were acquired in scan mode by means of electron impact (EI) MS. Cholesterol and FFAs were determined against d7Cholesterol and d31C16:0, respectively, with the MassHunter quantitative software (Agilent Technologies, CA, United States). Analyses were run in duplicate.

### Liquid chromatography-mass spectrometry

The chromatographic apparatus consisted of the 1260 Infinity II series LC system (Agilent Technologies, CA, United States). High resolution reversed phase liquid chromatography (RPLC) was perforfed with a Zorbax SB-C8 HT (2.1 × 100 mm, 1.8 µm p. s.) with a maximal operational backpressure at 600 Bar (Agilent Technologies, CA, United States). Lipid mixtures were eluted a gradient of (A) 5 mM ammonium formate in water, (B) methanol, (C) acetonitrile, (D) isopropanol. The elution program was as follows: A/B/C/D 60/28/8/40 at time 0 and held for 1 min, brought to A/B/C/D 1/70/20/9 in 10 min and held up to 20 min. The flow rate was maintained at 400 μL/min during the entire LC run. The column was thermostated at 60°C. The injection volume was 0.20 µL. The separation of polar lipids, e.g., phosphatidylcholines (PCs), phosphatidylethanolamins (PEs), and sphingomyelins (SMs), was performed on a HILIC stationary phase. The HaloHILIC column (2.1 × 50 mm, 2.7 µm p. s.) was purchased from Advanced Materials Technologies (PN 9281-1204, Wilmington, DE, US). The separation of lipids in HILIC mode was performed with a gradient of (A) 5 mM ammonium formate in water, (B) methanol, and (C) acetonitrile. The elution program was as follows: A/B/C 1/2/97 at time 0 and held for 1 min, brought to A/B/C 18/2/80 in 20 min and held up to 10 min. The flow rate was maintained at 400 μL/min during the entire LC run. The column was thermostated at 40°C. The injection volume was 1 µL. The mobile phases were filtered through 0.45 µm glass filters and continuously degassed under vacuum. The injector needle was washed with the mobile phase in the wash port during the LC runs.

Accurate mass measurements in full MS and auto MS/MS were conducted with a G6545B series LC-QTOF (Agilent Technologies, United States) equipped with a JetStream Technology electrospray interface (ESI) interface operating in both positive and negative ion mode. Analytes eluted from the LC system were introduced into the Q-TOF apparatus at the operating chromatographic flow rate (see chromatographic conditions). Nitrogen was used as the nebulizing and desolvation gas. The temperature and the flow of the drying gas temperature were 200°C, and 12 L/min, respectively. The temperature and the flow of the sheath gas were 350°C and 12 L/min, respectively. The nebulizer pressure was 40 psi. The capillary and the fragmentor voltage were 4,000 and 180 V, respectively. Full scan mass spectra were acquired in the positive and negative ion modes in the range from m/z 100 to m/z 1600. To enhance accurate mass measurement for the ion species a reference solution of two compounds with m/z 121.050873 and 922.009798, respectively, was vaporized in continuum in the spray chamber by means of a separate nebulizer. Analyses were performed in duplicate in each mode.

### Extraction of MS features

Molecular features, defined by an m/z, RT and signal intensity value, were extracted from the raw LCMS data files using the untargeted or the targeted batch recursive feature extraction in the MassHunter Profinder software (Agilent Technologies, United States). The features extracted were exported into a compound exchange format (CEF) reporting RT, the accurate mass and the absolute abundance for each entity to be processed in the subsequent chemometric analysis as previously reported (Ludovici et al., 2018; Singh et al., 2018).

### Data analysis (lipidomics)

Agilent Mass Profiler Professional (MPP version 15.1) was used to process the LCMS untargeted and targeted data. Retention times (RT) were aligned by setting a RT window of 0.6 min, whereas m/z binning was performed by setting windows at 10 ppm. Absolute abundance of each entity was normalised by the absolute abundance of the d31CerNS internal standard. Data were filtered by frequency of detection, which reflects the number of samples that presented particular features. A frequency filter was applied to data extracted from MPP and only entities present in 100% of samples belonging to at least one of the investigated groups were retained for the statistical analysis. Fold changes of filtered entities were compared between groups volcano plots in the MPP tools. Fold changes with *p* values <0.05 after Bonferroni’s correction were considered as significant. Identification of entities within the MPP workflow was performed based on the METLIN Metabolomics Database (http://metlin.scripps.edu/) and the Lipid Annotator software (Agilent Technologies, CA, United States). Quantitative assessment of cholesterol sulfate (CSO_4_) was performed with the deuterium labelled internal standard d7CSO_4_.

## Data Availability

The data presented in this study are deposited in the NIH GEO repository under GSE 232622.
