# Supplementary material for: RNA sequencing and lipidomics uncovers novel pathomechanisms in recessive X-linked ichthyosis
Source: Front Mol Biosci. 2023 Jun 7;10:1176802. doi: 10.3389/fmolb.2023.1176802 (PMC10285781; doi:10.3389/fmolb.2023.1176802)
Supplement: Supplementary file 2 [file DataSheet1.docx]

Supplementary Material

RNA Sequencing and Lipidomics Uncovers Novel Pathomechanisms in Recessive X-Linked Ichthyosis

**Farrell McGeoghan, Emanuela Camera, Miriam Maiellaro, Manasi Menon, Mei Huang, Priya Dewan, Stela Ziaj, Matthew P Caley, Michael Donaldson, Anton Enright, Edel A. O'Toole**


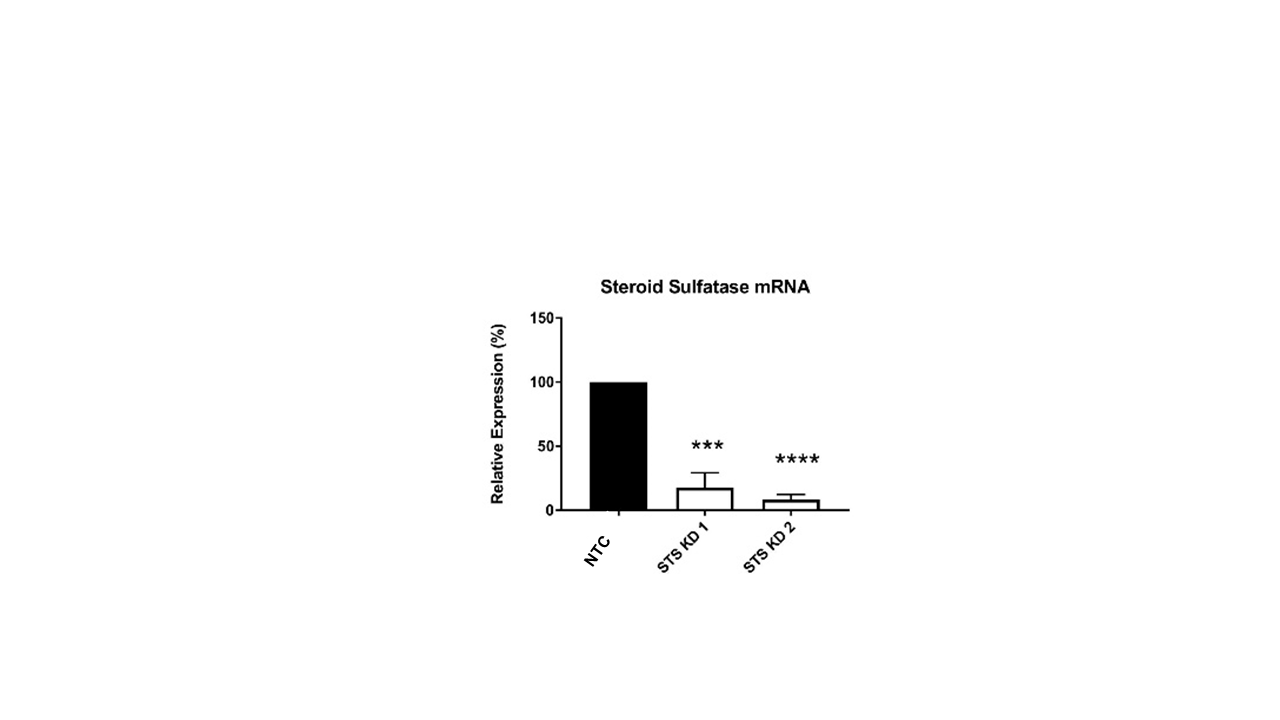


**Supplementary Figure S1A** Relative expression of STS mRNA in non-targeting control (NTC) transduced cells versus STS KD1 and STS KD 2 (n= 3 repeats of each). HPRT was used as a house-keeping control. *** P < 0.001. **** P< 0.0001.


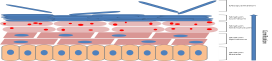


Media

Fibroblast

Keratinocyte

Epidermis

Collagen/Matrigel

Air-Liquid interface

Steel grid

Nylon mesh

**Supplementary Figure S1B** Schematic showing the organotypic skin model using keratinocytes and fibroblasts embedded in collagen/matrigel.


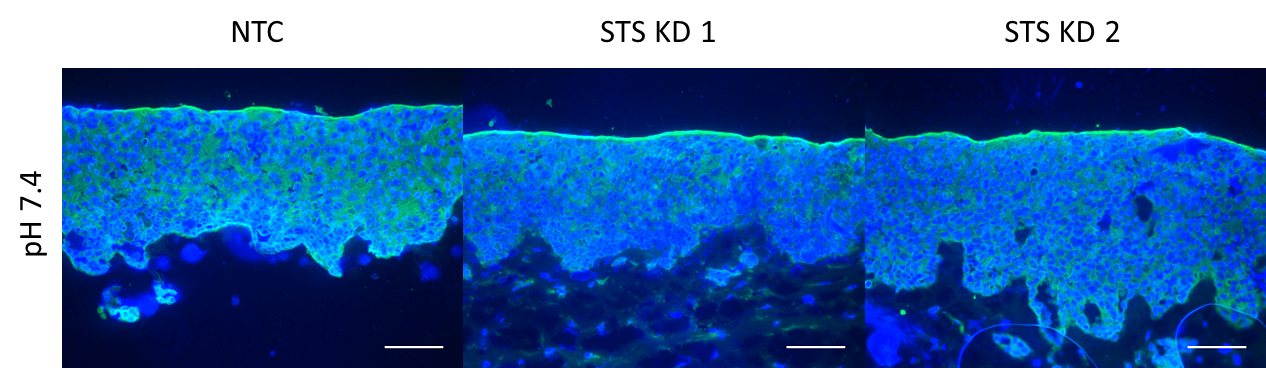


**Supplementary Figure S2** Transglutaminase activity assay at pH 7.4 showing activity throughout the epidermis in non-targeting control (NTC) which is reduced in STS KD1 and KD2. Scale bar = 100 um.


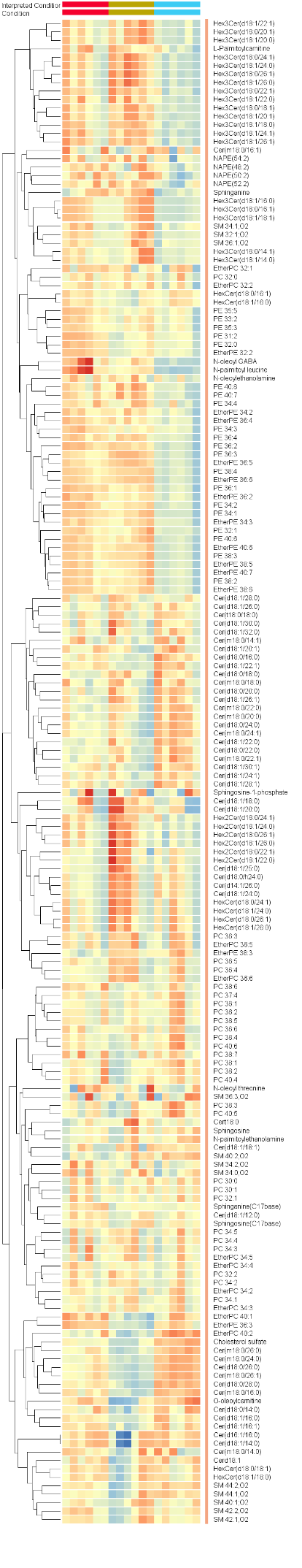


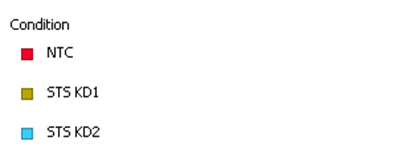


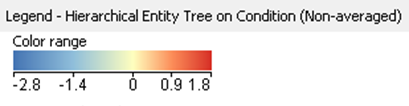


**Supplementary Figure S3** Hierarchical clustering tree of the lipid measured lipids NTC, STS KD1, and STS KD2. Supplementary Table 4 reports entities according their raw or normalized (log transformed) intensity values and cluster order in the dendogram.

**Supplementary Table S1**: **SMARTvector 2.0 lentiviral shRNA sequences**

| **Target Gene** | **shRNA #** | **Target Sequence** | **Catalogue #** | | |
| --- | --- | --- | --- | --- | --- |
| *STS* | shRNA_1 | TGACGAATCTTTGCAGAAC | SH-009602-02-10 | | |
| *STS* | shRNA_2 | TGAATGATCTCGTAGTTCC |  | SH-009602-03-10 |  |
|  |  |  |  |  |  |

##### Supplementary Table S2: Primary antibodies used for Western blotting (WB) and Immunofluorescence (IF).

| **Antigen** | **Clone** | **Species** | **Source** | **WB Dilution** | **IF Dilution** |
| --- | --- | --- | --- | --- | --- |
| ACER1 | HPA042506 | Rabbit | Atlas | 1:500 | 1:100 |
| ALDH1A1 | ab23375 | Rabbit | Abcam (UK) | 1:1000 | 1:100 |
| ALDH3A1 | ab76976 | Rabbit | Abcam (UK) | - | 1:100 |
| GAPDH | - | Rabbit | Abcam (UK) | 1:1000 | - |
| OXTR | ab181077 | Rabbit | Abcam (UK) | 1:1000 | - |
| OXTR | Ab87312 | Goat | Abcam (UK) | - | 1:100 |
| SPHK1 | Ab37980 | Rabbit | Abcam (UK) | - | 1:100 |
| Transglutaminase 1 | HPA040171 | Rabbit | Sigma | - | 1:100 |
| UGCG | ab124296 | Rabbit | Abcam (UK) | 1:1000 | 1:100 |
| DSG1 | 62/Desmoglein | Mouse | BD |  | 1:100 |
| KLK5 | ab7283 | Rabbit | Abcam |  | 1:100 |

ACER1, Alkaline Ceramidase 1; ALDH1A1, Aldehyde Dehydrogenase 1 Family Member A1; ALDH3A1, Aldehyde Dehydrogenase 3 Family Member A1; GAPDH, Glyceraldehyde-3-Phosphate Dehydrogenase; OXTR, Oxytocin Receptor; SPHK1, Sphingosine Kinase 1; UGCG, UDP-Glucose Ceramide Glucosyltransferase; DSG1, Desmoglein 1; KLK5, Kallikrein 5.

##### Supplementary Table S3: Primer sequences used for qPCR.

| **Primer name** | **Sequence (5’ – 3’)** | **Target gene** |
| --- | --- | --- |
| HPRT Forward | GAAGAGCTATTGTAATGACC | *HPRT* |
| HPRT Reverse | GCGACCTTGACCATCTTTG | *HPRT* |
| STS Forward | AACTCACTCAGCACCTGGCA | *STS (steroid sulfatase)* |
| STS Reverse | GGGAGGAAGACCAGCCTCTT | *STS (steroid sulfatase)* |
